# Initial experience in treating lung cancer with helical tomotherapy

**DOI:** 10.2349/biij.3.1.e2

**Published:** 2007-01-01

**Authors:** S Yartsev, AR Dar, C Woodford, E Wong, G Bauman, J Van Dyk

**Affiliations:** 1 London Regional Cancer Program, London Health Sciences Centre, London, Ontario, Canada; 2 The University of Western Ontario, London, Ontario, Canada

**Keywords:** Image-guided adaptive radiotherapy, helical tomotherapy, megavoltage CT, lung cancer

## Abstract

Helical tomotherapy is a new form of image-guided radiation therapy that combines features of a linear accelerator and a helical computed tomography (CT) scanner. Megavoltage CT (MVCT) data allow the verification and correction of patient setup on the couch by comparison and image registration with the kilovoltage CT multi-slice images used for treatment planning. An 84-year-old male patient with Stage III bulky non-small cell lung cancer was treated on a Hi-ART II tomotherapy unit. Daily MVCT imaging was useful for setup corrections and signaled the need to adapt the delivery plan when the patient’s anatomy changed significantly.

## INTRODUCTION

Helical tomotherapy is a radiotherapy technique that combines the geometry of a diagnostic helical Computed Tomography (CT) scanner with the capability to deliver highly conformal radiation dose distributions in an intensity-modulated fashion [1,2]. The same linac is also used for obtaining MVCT images prior to actual daily fractionated treatment. There are at least two major benefits provided by such imaging: i) a correction of setup errors [3-5] which is especially important in cases where rigid immobilisation is difficult or internal motion is common (i.e. extracranial sites such as thorax/abdomen, some lung cancer patients have difficulty in keeping their arms up even in a vac-loc immobilisation device, mostly because of arthritis in the shoulders) and ii) a modification of the treatment itself based on information obtained from MVCT images. The latter feature, often referred to as image-guided adaptive radiotherapy, has already been discussed in the literature [6-10]. Ramsey *et al*. have done a retrospective treatment planning study and concluded that weekly plan adjustment of tomotherapy plans may reduce the absolute volume of ipsilateral lung receiving 20 Gy by 17-23% in lung cancer patients [6,10]. Kupelian *et al.* have observed a gradual reduction of the gross tumour volume (GTV) ranging from 0.6-2.3% per day in their non-small-sell lung cancer patients treated on the tomotherapy unit [7-9]. In this communication, we describe a clinical case where treatment plan was modified after 22 fractions and various options to perform adaptive radiotherapy were discussed based on information provided by a daily MVCT imaging. In particular, we assess the clinical significance of modifications made to radiation delivery plans prompted by changes in GTV revealed by MVCT images during fractionated lung cancer treatment on the tomotherapy unit.

## METHODS AND MATERIALS

**Figure 1:**
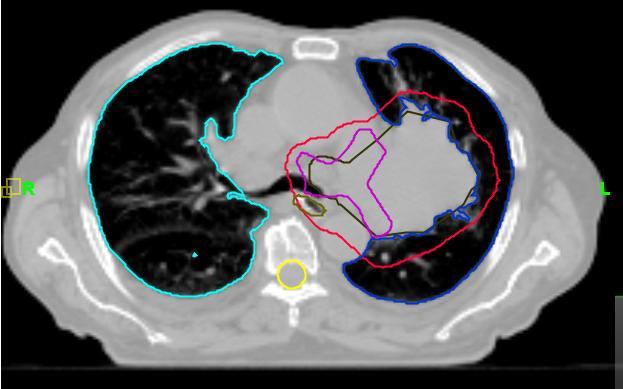
Axial slice of kVCT study made 17 days before treatment start date. This was used for the creation of treatment Plan 1.

**Figure 2:**
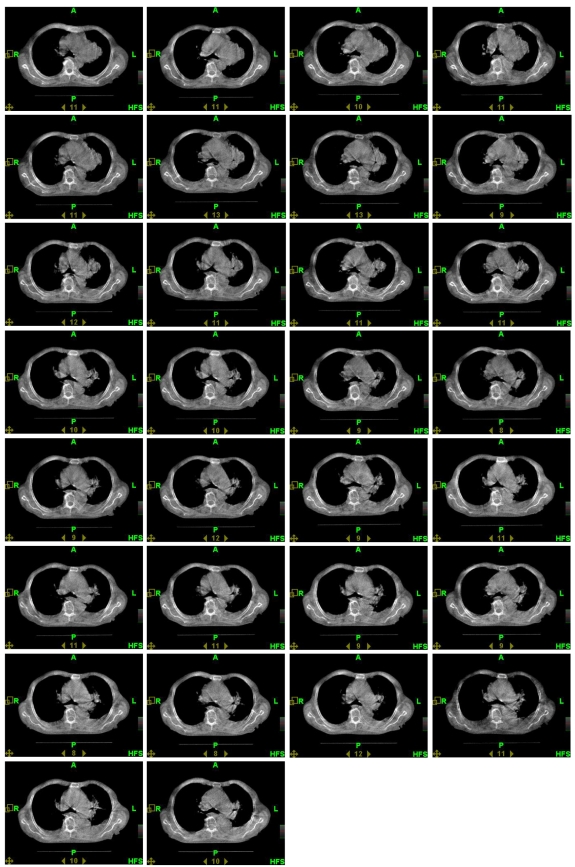
30 images corresponding to 30 fractions of treatment. Reduction of tumour volume is clearly observed.

**Figure 3:**
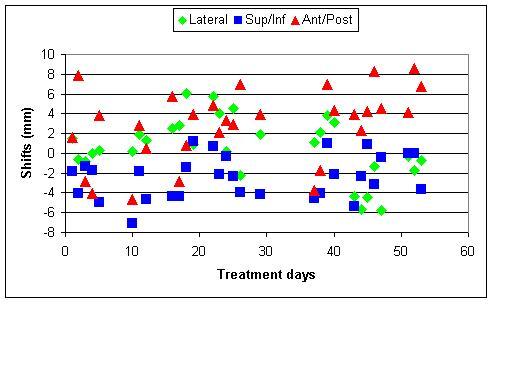
Daily setup shifts determined from MVCT/kVCT registration.

**Figure 4:**
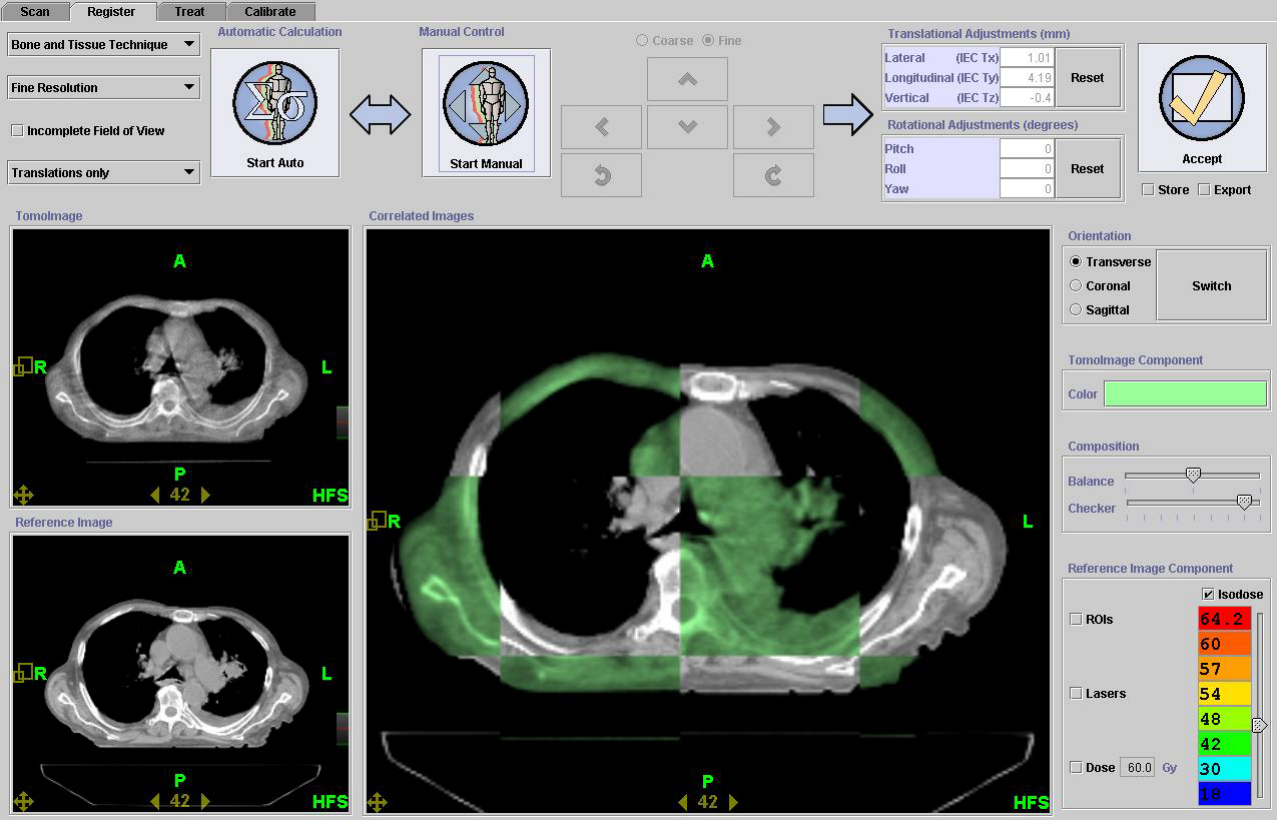
Registration screen on the operating station used for automatic and/or manual alignment of the MVCT images (upper left and green colour on the central frame) with the planning kVCT images (lower left and white colour on the central frame).

**Figure 5:**
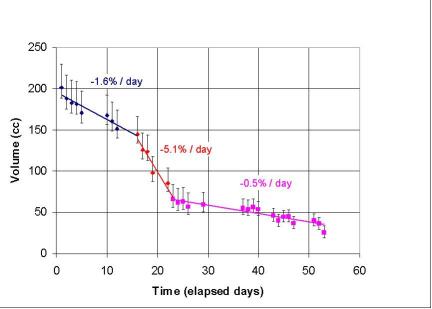
GTV reduction during the treatment showing three distinct phases.

**Figure 6:**
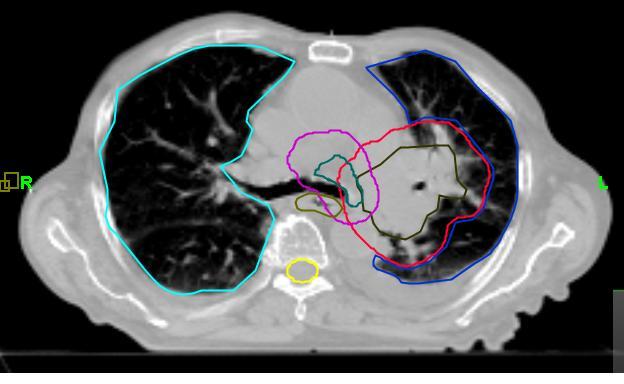
Axial slice of kVCT study made 39 days after treatment start date. This study, with the 3D margin around GVT of 1.2 cm was used for the creation of treatment Plan 2.

**Figure 7:**
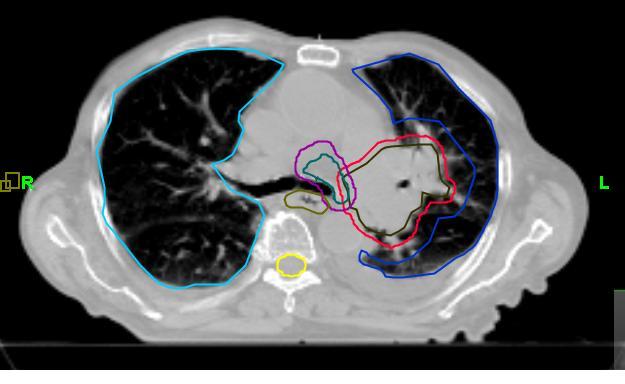
Axial slice of kVCT study made 39 days after treatment start date. This study with the 3D margin around GVT of 0.5 cm was used for the creation of treatment Plan 3.

An 84-year-old male patient presented with symptomatic left upper lobe and hilar mass, Stage III bulky non-small cell lung cancer without atelectasis. A kilovoltage CT (kVCT, Philips Brilliance Big Bore, 3 mm slice thickness, 120 kVp, 300 mAs/Slice) image (Figure 1) was taken 17 days before the start of treatment and the radiation oncologist (ARD) outlined two targets (gross tumour volume (GTV) and mediastinal nodes) and the following sensitive structures: lungs, esophagus, spinal cord, heart. Planning target volumes (PTV) were created by a 12 mm 3D margin around GTV (PTV Lung) and around mediastinal nodes (PTV Nodes). Doses of 60 Gy to PTV Lung and 50 Gy to PTV Nodes in 30 fractions were prescribed. The helical tomotherapy plan based on this anatomy (plan 1: field slice thickness 2.5 cm, pitch 0.286, expected beam-on time 465 s per fraction) was approved for treatment. All treatments were preceded by daily MVCT imaging (Figure 2). The MVCT study was used for two reasons: a) to correct for inter-fraction changes of the patient’s position (Figure 3) on the couch by co-registration of the MVCT study with the kVCT study used for treatment (Figure 4), and b) to assess variations in tumour size and/or positioning inside the patient. MVCT images for all treatment days were transferred to the planning station and GTV were contoured on all of them. The variation of the GTV with time is presented in Figure 5. The error bars were determined by outlining the maximum and minimum imaginable GTVs on MVCT studies made on Day 1 and Day 43 of treatment. After 15 fractions, the MVCT images showed a tumour size reduction of 70%. However, the radiation oncologist decided to continue the treatment according to Plan 1 until delivery of 44 Gy in 22 fractions to ensure sterilisation of a sub-clinical microscopic disease of the initial target. A repeat kVCT study (Figure 6) was performed and a new plan with new structure outlines to reflect the anatomy changes (Plan 2: beam-on time 388 s) was applied for the remaining eight fractions. To test the clinical significance of the margin choice we have created another set of target outlines (Figure 7) with a 0.5 cm margin around GTVs.

Subsequent follow-ups after four and eight months showed (by physical examination and diagnostic kVCT with contrast) moderate radiation pneumonitis (Grade 2) that resolved with fibrosis. The patient follow up continued for 11 months with no evidence of cancer recurrence.

## RESULTS AND DISCUSSION

**Figure 8:**
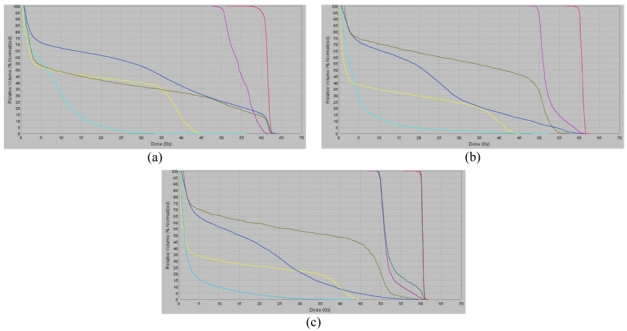
(a) Dose volume histogram of Plan 1. Colour code: black: GTV; red: PTV; violet: PTV Nodes; blue: left lung; light blue: right lung; yellow: spinal cord; brown: oesophagus, (b) dose volume histogram of Plan 2, (c) dose volume histogram of Plan 3.

**Figure 9:**
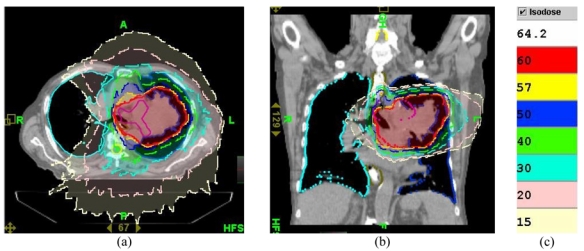
Dose distribution ((a) axial and (b) sagittal views) as calculated by Plan 1 on the kVCT image used for this plan. (c) Colour code for isodose lines.

**Figure 10:**
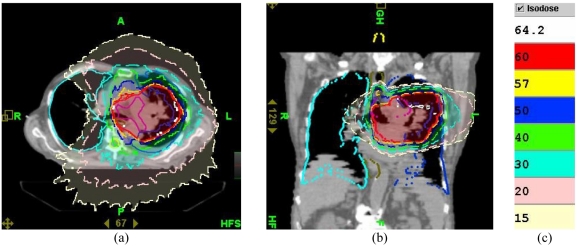
Dose distribution ((a) axial and (b) sagittal views) which would have been produced by radiation fluence of Plan 1 in the patient on 39th day of the treatment. (c) Colour code for isodose lines.

**Figure 11:**
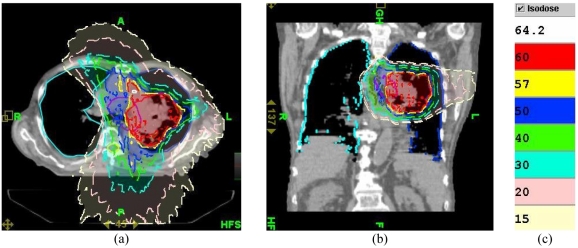
Dose distribution ((a) axial and (b) sagittal views) produced by radiation fluence of Plan 2 in the patient on 39th day of treatment. (c) Colour code for isodose lines.

**Figure 12:**
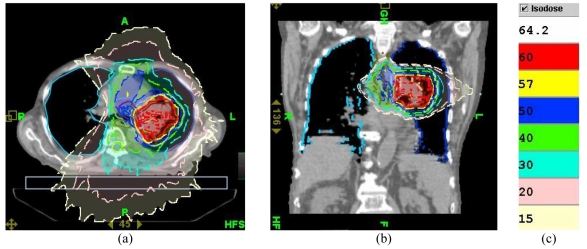
Dose distribution ((a) axial and (b) sagittal views) which would have been produced in the patient on 39th day of treatment if radiation fluence of Plan 3 is used. (c) Colour code for isodose lines.

Sensitive structures tolerance dose criteria [11] were met in all three plans as shown in the dose-volume histograms (DVH) for plans 1, 2, and 3 in Figures 8 (a), (b), and (c), respectively. The full dose was delivered to the primary target and the nodes without any side effects. The PTV was reduced to 769 cm^3^ in Plan 1 to 386 cm^3^ in Plan 2 and to 193 cm^3^ in Plan 3. The calculated mean lung dose was 16.3 Gy according to Plan 1, 10.4 Gy in Plan 2, and 7.9 Gy in Plan 3. The planned dose distribution according to Plan 1 is shown in Figure 9. We note high conformity of the 60 Gy isodose line to the PTV contour. If this plan would have been delivered 39 days after the start of the treatment when the tumour size has been significantly reduced (Figure 5), a large region of healthy lung tissue would have been irradiated as shown in Figure 10. However, Plan 2 (adapted for the changing tumour size) delivers the prescription dose quite comfortably on Day 39 (Figure 11), with no irradiation to healthy lung tissue due to a lower mean lung dose of 10.4 Gy. Clinically, there is a possibility to reduce the margin around GTV after delivery of 44 Gy because the microscopic disease spread which defines the margin of PTV to CTV should be eliminated by this dose. In this case, we can use quite a tight margin for generating the PTV, because set-up errors are under control by the registration process shown in Figure 4. Our calculations with the plan with such a tighter margin (Plan 3) gave a dose distribution presented in Figure 12 with superior lung tissue sparing and a mean lung dose of only 7.9 Gy.

In this case, there were at least three phases in tumour reduction, with initial phase of ca. 10 fractions (or 20 Gy with delivery of 2 Gy per fraction) when tumour reduction of 1.6% per day was noted. After this dose there was a rather rapid reduction in tumour size, of more than 5% per day which continued for three weeks until 40 Gy was administered. Finally, there was a slow reduction in tumour volume at 0.5% per day which probably continued when the treatment was finished at 60 Gy. The possible mechanisms for such a three phase reduction include an initial accumulation of radiation damage in Phase 1 before a radiosensitive Phase 2 of rapid reduction due to re-oxygenation occurs, while in Phase 3 cell death is counterbalanced by repopulation. Such a hypothesis requires verification in larger number of patients and correlation with histology or non-invasive information about tumour metabolism by PET/SPECT.

## CONCLUSION

The combined usage of kVCT and MVCT studies allowed high quality kVCT images for precise radiation dose calculations on tomotherapy planning station for both initial and adapted plans, while MVCT scans were used for accurate patient setup and monitoring of tumour evolution. MVCT has sufficient image quality for verification of daily treatment delivery allowing for plan adaptation according to tumour response. It can be used to quantitatively measure tumour response during radiotherapy. Furthermore, daily imaging provides data necessary for an optimal choice of GTV to PTV margin based on values for setup corrections and tumour dynamics.
